# EHMTI-0083. Can patients manage without methysergide?

**DOI:** 10.1186/1129-2377-15-S1-G29

**Published:** 2014-09-18

**Authors:** R Peatfield

**Affiliations:** 1 Princess Margaret Migraine Clinic, Charing Cross Hospital, London, UK

## Background

Methysergide has been the prophylactic drug of last resort at the Princess Margaret Migraine Clinic for many years. In Boston I reviewed the records of 38 patients, 27 with migraine and 11 cluster headache, including 11 who had been taking it continuously, in some cases for as long as ten years. The distribution of methysergide ceased in May 2013, and supplies dwindled in Britain during the autumn of 2013.

I have reviewed the 2013-4 records of these 11 patients, as well as an additional 4 (3 migraine and 1 cluster headache) who had started methysergide under our supervision during the early months of 2013. Two of the 13 migraine patients had remitted spontaneously, and in a further one the drug seemed to have lost efficacy; the remaining 10 and both the cluster patients were worse.

The European Medicines Agency have acknowledged the value of methysergide1, recommending that all patients are registered so that screening tests for fibrotic complications are undertaken regularly. The British Association for the Study of Headache will be arranging this; no doubt comparable arrangements will be set up in other countries. The British company Amdipharm holds a distribution licence for methysergide covering most of Europe; they hope to restore it to the market soon.

## Conclusions

These findings suggest that there will be always be a significant number of patients who would benefit from the drug.

No conflict of interest.
